# Difficulty Breathing or Just a Case of the Nerves? Incidental Finding of Primary Pleural Schwannoma in a COVID-19 Survivor

**DOI:** 10.7759/cureus.17511

**Published:** 2021-08-27

**Authors:** Daania Shoaib, Muhammad N Zahir, Saqib R Khan, Adnan A Jabbar, Yasmin A Rashid

**Affiliations:** 1 Internal Medicine, Aga Khan University Hospital, Karachi, PAK; 2 Medical Oncology, Aga Khan University Hospital, Karachi, PAK

**Keywords:** primary pleural schwannoma, covid-19, computed tomography, incidental radiological finding, peripheral nerve sheath tumor, schwannoma, pleural schwannoma

## Abstract

Schwannoma is a rare tumor that arises from the Schwann cells, which are specialized, myelin-producing cells of the peripheral nerve sheaths. As anatomic logic would dictate, these masses commonly occur in the skull base, cerebellopontine angle, and posterior spinal roots. Of this already rare entity, rarer still are the pleural schwannomas, representing approximately 1-2% of thoracic tumors. These tumors commonly affect adults with a propensity for the third and sixth decades of life and a comparative male predilection. Schwannomas are benign, indolent, and follow an asymptomatic course. As such, they often come to light incidentally.

Here we report a case of primary pleural schwannomas in a 68-year-old female, found incidentally on a CT scan of the chest. To the best of our knowledge and literature review, no other similar case has been reported in our country, Pakistan. Around three weeks before her presentation, she was diagnosed with COVID-19. Her infection had run a mild course with quick recovery without the need for any hospitalization. Therefore, the manifestation of shortness of breath after resolution of all other symptoms prompted a further workup. Radiographic chest x-ray revealed an incidental finding of a large right upper lobe lung mass, slightly impinging on the trachea. This was followed by a chest CT scan at our radiological imaging facility, which showed a large, well-encapsulated, right upper lobe lung mass in the paraspinal apical location. She then underwent an image-guided biopsy of the aforementioned mass, pathological analysis of which was suggestive of a benign peripheral nerve sheath tumor (PNST) arising from the pleura (pleural schwannoma). She underwent right posterolateral thoracotomy with uneventful complete surgical removal of the pleural-based lung mass. Postoperative investigations included a chest x-ray that showed interval complete resection of the mass. Currently, she is asymptomatic and her clinical condition has improved with the successful resumption of her daily routine.

Physicians thus need to keep pleural schwannomas in mind as a probable diagnosis of intrathoracic tumors. Indolent and asymptomatic, they are very amenable to surgical resection with little to no chances of recurrence in the long term. However, these patients should be closely followed with repeat imaging studies when symptomatic.

## Introduction

Schwannoma, also called neurilemmomas, are rare tumors that arise from the Schwann cells, which are specialized, myelin-producing cells of the peripheral nerve sheaths. They are usually well-circumscribed, encapsulated, and almost always solitary [1]. Furthermore, these tumors histopathologically reflect their origin, being closely related to the normal Schwann cells [2].

As anatomic logic would dictate, these masses commonly occur in the skull base, cerebellopontine angle, and posterior spinal roots [1]. However, literature does report other locations such as the neck, chest wall, and cavity; with up to 75% arising from the posterior mediastinum, usually in association with paraspinal nerves and soft-tissue sites [2,3].

Of an already rare entity, rarer still are the pleural schwannomas, representing approximately 1-2% of thoracic tumors [1,4] and arise from the autonomic nerve fiber sheaths present on the pleural surface of the lung [5,6]. These tumors commonly affect adults with a propensity for the third and sixth decades of life, and a comparative male predilection [1,4]

Schwannomas are benign, indolent, and follow an asymptomatic course. As such, they often come to light incidentally [4]. Although malignant schwannomas do exist, they are exceedingly rare with a ratio of 1:11 when contrasted with their benign counterparts [7].

Here we report a case of primary pleural schwannomas in a 68-year-old female, found incidentally on a CT scan of the chest. To the best of our knowledge and literature review, no other similar case has been reported in Pakistan.

## Case presentation

A 68-year-old woman, resident of Karachi, Pakistan, with known comorbidity of type-two diabetes mellitus, presented with complaints of mild but persistent shortness of breath on exertion, for one to two weeks. She never smoked and had no history of substance abuse.

Around three weeks before her presentation, she was diagnosed with COVID-19. Her infection had run a mild course with quick recovery without the need for any hospitalization. Therefore, the manifestation of shortness of breath after resolution of all other symptoms prompted a further workup.

**Figure 1:**
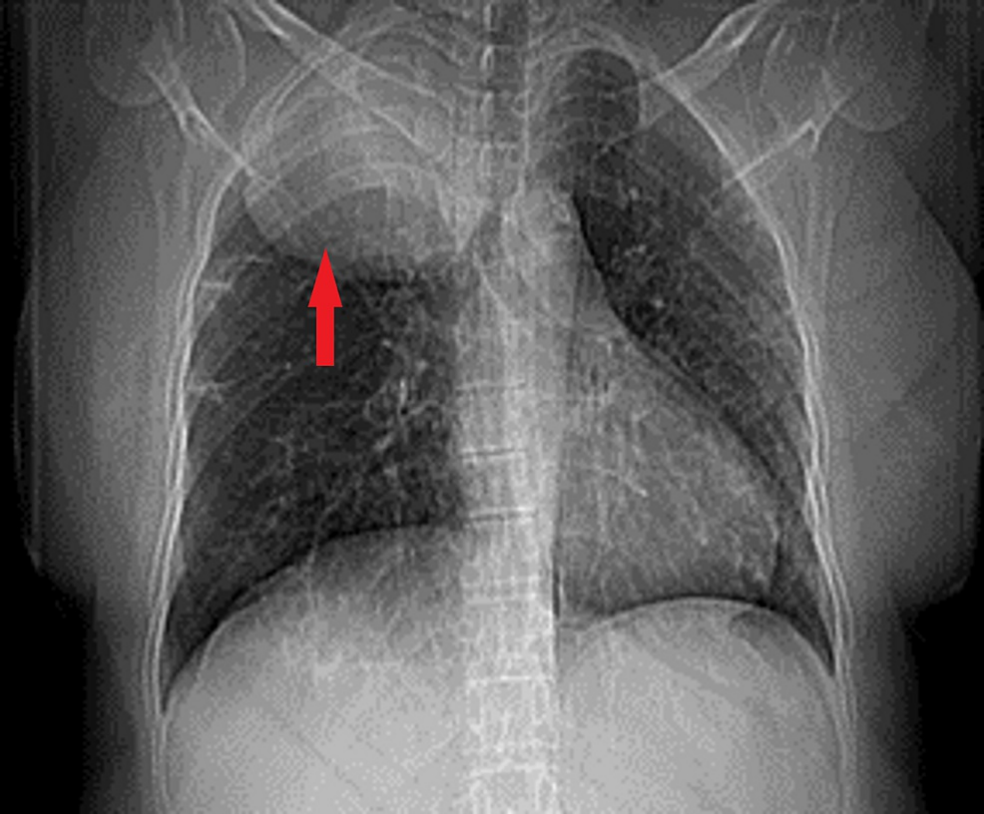
Chest x-ray on presentation showing a large right upper lung lobe mass, slightly impinging on the trachea (marked with red arrow).

On her clinic visit, she reported no neurological or any other systemic symptoms. On general physical examination, she was of average height and build and her vital parameters were within reference ranges. Her systemic examination revealed slightly decreased breath sounds on chest auscultation over the right upper lung field, with the remainder of the systemic examination being unremarkable. As she was symptomatic, she was admitted with the impression of post-COVID-19 sequelae. Relevant blood investigations were performed and all were within normal ranges. Radiographic chest x-ray revealed an incidental finding of a large right upper lobe lung mass, slightly impinging on the trachea (Figure 1).

**Figure 2:**
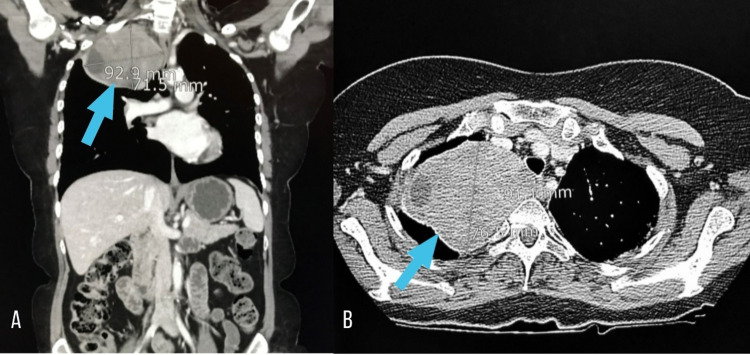
CT scan – Right paraspinal, apical mass with internal cystic areas, approximately 9.5 x 7cm, seen in coronal (A) and axial (B) views, respectively

This was followed by a CT scan of the chest at our radiological imaging facility, which showed a large, well-encapsulated, right upper lobe lung mass in the paraspinal apical location, measuring 95x70 mm and forming an obtuse angle with the right-sided pleura (Figure 2). A contrast-enhanced CT scan of the abdomen and pelvis was later performed at our institution which did not show any significant finding. 

**Figure 3:**
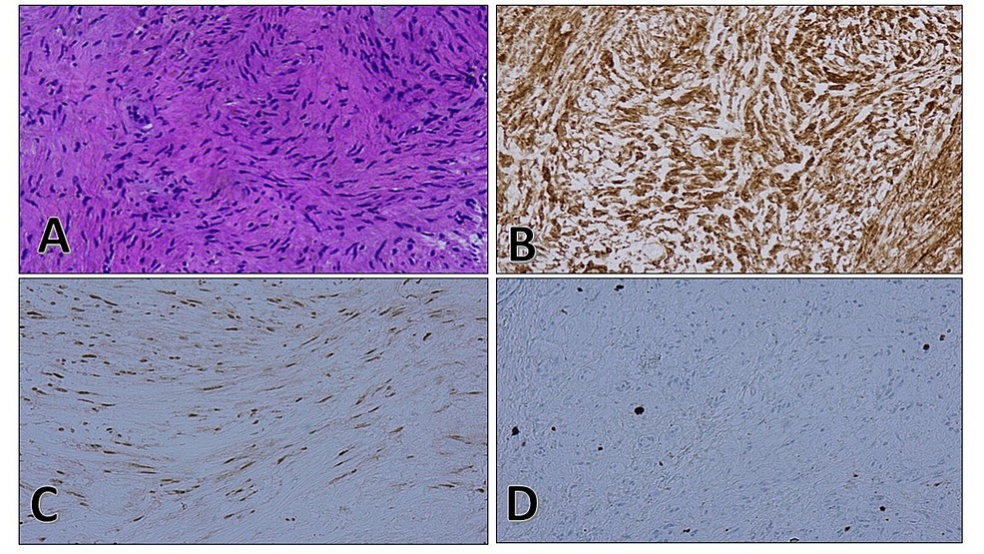
(A) Hematoxylin and eosin (H and E) stain showing spindle cells and wavy nuclei with no polymorphism. (B) IHC staining showing strong positivity for S100. (C) IHC staining showing Sox10 patchy positive. (D) IHC staining of Ki67 shows low proliferative index IHC: Immunohistochemistry

Based on the above findings, she underwent an image-guided biopsy of the aforementioned mass, pathological analysis of which showed a spindle cell lesion with low cellularity and wavy nuclei with focal palisading. The immunohistochemical stains showed a predominant positive reactivity for S-100 and Sox-10. All of these features were consistent with the diagnosis of a benign peripheral nerve sheath tumor (PNST), arising from the pleura, a pleural schwannoma. The patient was discussed in the multidisciplinary team (MDT) meeting and surgical resection of the mass was recommended. After a detailed discussion with the patient and informed consent, she underwent right posterolateral thoracotomy with uneventful complete surgical removal of the pleural-based lung mass. Operative findings revealed a hard, 10x10cm posterior mediastinal mass. (Figure 3)

**Figure 4:**
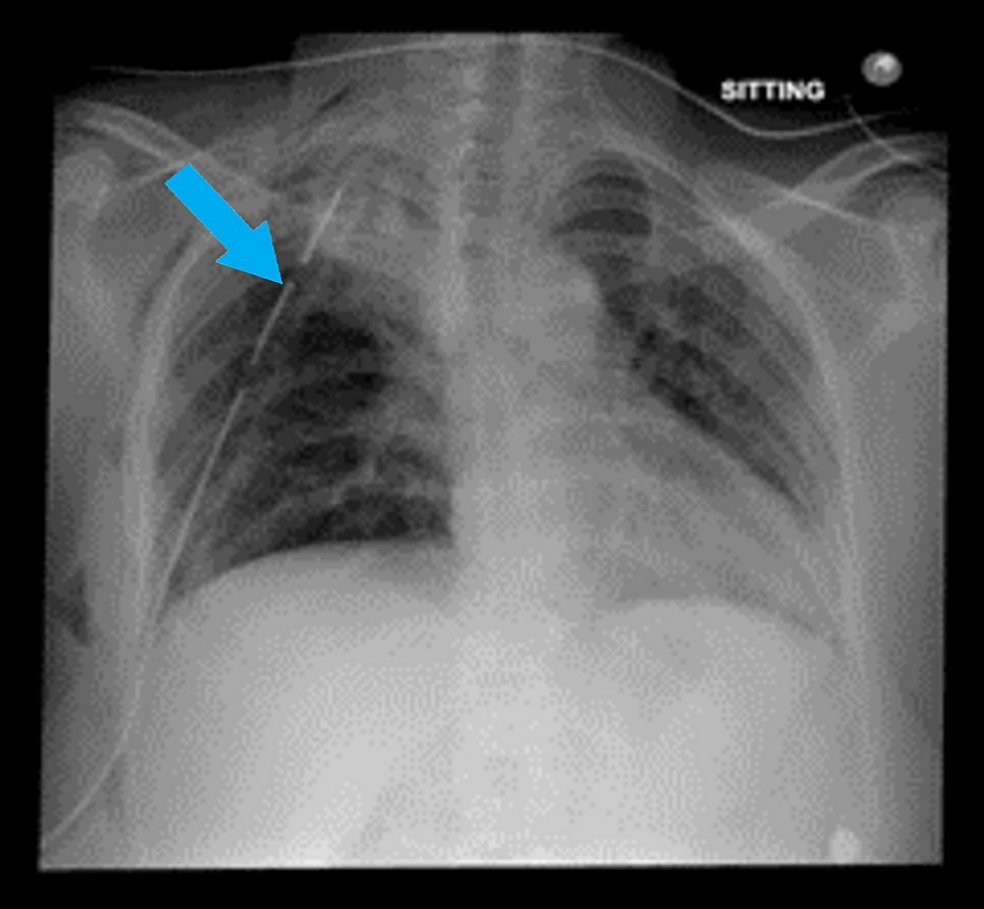
Chest x-ray on postoperative day one shows interval complete resection of the mass. A right-sided chest tube is also seen in situ.

Postoperative investigations included a chest x-ray, which showed interval complete resection of the mass (Figure 4). She is further planned for a follow-up CT scan at three months interval post-surgical resection of her lung mass. She has been assessed as an outpatient at the 30-day and 45-day mark. Recovery is well underway and she is asymptomatic with an improved clinical condition and successful resumption of her daily routine.

## Discussion

Mostly benign, PNSTs represent at least 85-90% of clinically symptomatic cases, and an even larger ratio in subclinical cases [8]. These can be further classified as schwannomas, neurofibromas, and perineuriomas based on their histological features [3].

Pleuropulmonary PNSTs tend to show the same diversity as their counterparts in more common locations and can occur as endobronchial, pleural, or parenchymal locations [3]. Even though primary pleural schwannomas are rare, accounting for 1%-2% of all thoracic tumors, they are a common occurrence within the category of pleuropulmonary PNSTs. In a study involving 75 cases of intrathoracic PNSTs conducted by Boland et al. in 2015, 21 cases of benign pleuropulmonary PNSTs were identified, out of which schwannomas were found to comprise roughly 60% [3]. Similar results were published in a comprehensive study carried out in Japan, elaborating on 50 years’ worth of thoracic neurogenic tumors, whereby 43% of all adult intrathoracic PNSTs were reported as schwannomas [9]. As evident from this data, schwannomas seem to lead the board where they most commonly form pleural-based masses [3].

Pleural schwannomas commonly affect adults between their third and sixth decades with males more commonly affected than females. These tumors are rather indolent and grow at an exceptionally slow rate over many years and as such rarely produce any symptoms. Discovery is often incidental on imaging studies or bronchoscopies performed for other indications. Studies have shown that up to 65-85% of pleural schwannomas are asymptomatic [3,9]. When symptoms occur; they act as the catalyst for seeking medical care. The most common symptoms that have been reported are shortness of breath, cough, hemoptysis, and obstructive pneumonia. Generally, these symptoms may be seen when a tumor grows large enough to start affecting vital adjacent structures. Our patient, despite being asymptomatic for many years, only began to show symptoms when her pulmonary function reserve became slightly impaired by a COVID-19 infection.

Rarely, benign schwannomas may present with pleural effusions or blood-stained exudates. A recent study demonstrated an incidence of 2% for effusions in benign schwannomas [10]. These effusions can be large in volume and often blood-stained and should not serve as an automatic indicator of malignancy. The possible etiology behind blood-stained effusions has been postulated as spontaneous tumor hemorrhage or rupture [4,11,12]

Benign PNSTs usually arise sporadically. However, associations with certain genetic conditions have been observed in the literature. For instance, multiple neurofibromas indicate neurofibromatosis type 1 (NF1) (Von Recklinghausen disease) and when multiple schwannomas are present, this may signify neurofibromatosis type 2 (NF2) and schwannomatosis. Malignant schwannomas have been noted to have a link to NF1; Valeyrie-Allanore et al. noted that approximately 50% of adult patients with malignant neurogenic tumors were reported to have this disease [13]. Comparatively, malignant lesions tend to be more symptomatic, often causing pain, severe dyspnea, pericardial effusions, and very commonly pleural effusions.

Diagnosis of pleural schwannomas can sometimes prove to be a challenge. Advanced imaging modalities may often fail to return a definitive answer. However, they do serve to steer the conversation towards considering schwannomas. Imaging proves helpful in differentiating between isolated pleural lesions with pleural metastases, lipomas, or fibrous tumors. Kransdorf demonstrated that CT scans help to present features suggestive of schwannomas [14]. These usually are discrete, oval lesions with well-defined margins, with iso or hypoattenuation in comparison to thoracic wall muscles [15]. Malignant schwannomas may demonstrate similar features but will almost always be associated with pleural nodules, effusions, and metastatic pulmonary nodules. MRI can help to delineate the extent of vascular involvement of the malignant tumor [5]. Schwannoma shows a wide variation in standardized uptake values (SUVs) in positron emission tomography (PET) scans, making a distinction of schwannomas from a malignant PNST or other malignant tumors that are much more difficult [16]. The reason for this high uptake in a benign entity such as schwannomas remains unclear.

Conclusive diagnosis rests on histopathological analysis and subsequent immunohistochemical staining, requiring a tissue specimen [6]. The most classical marker for schwannomas remains the S-100 stain, which is usually strong and diffuse, helping to clarify ambiguities in diagnosis. Other histological features seen very commonly are nuclear palisading with elongated and wavy contours and Antoni A and B areas (alternating hyper and hypocellular areas respectively) [2,3].

The gold standard of treatment for pleuropulmonary tumors remains surgical excision. Conservative observation has little to no justification, as primary surgical removal serves a manifold purpose, providing definitive diagnosis as well as therapy. Depending on the characteristics of the tumor, a surgical approach could involve either a minimally invasive procedure or an open thoracotomy; the precise indications of either of which are beyond the scope of this paper [2].

Recurrence in cases of surgically resected schwannomas is a rare occurrence; accounting for less than 5% [17]. Neither of the studies that have dealt with these tumors has reported a recurrence or metastasis. Sometimes a local recurrence may occur with benign PNSTs, which is not necessarily indicative of malignant potential or behavior [18]. Currently, because of the limited data available for the management of pleural schwannomas, the mainstays of the treatment are surgical resection followed by observation with imaging studies when clinically indicated. 

## Conclusions

Pleural schwannomas should be kept in mind as a probable diagnosis of intrathoracic tumors. Indolent and asymptomatic, they are very amenable to surgical resection with little to no chances of recurrence in the long term. However, these patients should be closely followed with repeat imaging studies when symptomatic. 
